# Translational feasibility and efficacy of nasal photodynamic disinfection of SARS-CoV-2

**DOI:** 10.1038/s41598-022-18513-0

**Published:** 2022-08-24

**Authors:** Layla Pires, Brian C. Wilson, Rod Bremner, Amanda Lang, Jeremie Larouche, Ryan McDonald, Joel D. Pearson, Daniel Trcka, Jeff Wrana, James Wu, Cari M. Whyne

**Affiliations:** 1 University Health Network, Toronto, Canada; 2 Faculty of Medicine, University of Toronto, Toronto, Canada; 3 Lunenfeld-Tanenbaum Research Institute, Mount Sinai Hospital, Toronto, Canada; 4 Roy Romanow Provincial Laboratory, Saskatchewan Health Authority, Regina, Canada; 5 Holland Bone and Joint Program, Sunnybrook Research Institute, 2075 Bayview Avenue S620, Toronto, ON M4N3M5 Canada

**Keywords:** Viral infection, Translational research

## Abstract

The lack of therapeutic options to fight Covid-19 has contributed to the current global pandemic. Despite the emergence of effective vaccines, development of broad-spectrum antiviral treatment remains a significant challenge, in which antimicrobial photodynamic therapy (aPDT) may play a role, especially at early stages of infection. aPDT of the nares with methylene blue (MB) and non-thermal light has been successfully utilized to inactivate both bacterial and viral pathogens in the perioperative setting. Here, we investigated the effect of MB-aPDT to inactivate human betacoronavirus OC43 and SARS-CoV-2 in vitro and in a proof-of-principle COVID-19 clinical trial to test, in a variety of settings, the practicality, technical feasibility, and short-term efficacy of the method. aPDT yielded inactivation of up to 6-Logs in vitro, as measured by RT-qPCR and infectivity assay. From a photo-physics perspective, the in vitro results suggest that the response is not dependent on the virus itself, motivating potential use of aPDT for local destruction of SARS-CoV-2 and its variants. In the clinical trial we observed variable effects on viral RNA in nasal-swab samples as assessed by RT-qPCR attributed to aPDT-induced RNA fragmentation causing falsely-elevated counts. However, the viral infectivity in clinical nares swabs was reduced in 90% of samples and undetectable in 70% of samples. This is the first demonstration based on quantitative clinical viral infectivity measurements that MB-aPDT is a safe, easily delivered and effective front-line technique that can reduce local SARS-CoV-2 viral load.

## Introduction

Over the past two and a half years, there has been an urgent and ongoing challenge to find new and effective options to treat and prevent the spread of the SARS-CoV-2 virus. The lack of specific and efficient antiviral approaches has contributed to the current pandemic, with over six million deaths worldwide. Despite rapid development of effective vaccines, new mutations continue to emerge and broad-spectrum antiviral therapies are needed to lower the risk of immune escape by SARS-CoV-2 variants as well as future respiratory pathogens^1^.

SARS-CoV-2 RNA has been detected in aerosol particles of a range of sizes exhaled during normal tidal breathing^2,3^. Aerosol modeling suggests that the highest multiplicity of infection per unit tissue surface area for this virus occurs in the nose^4^. In addition, the nasal epithelium has the highest expression of ACE2, the primary SARS-CoV-2 binding site. At the early stages of infection, the highest virus titer has been measured in the nose compared to elsewhere in the respiratory tract^5–7^. These studies highlight the central role played by the nose in SARS-CoV-2 initial infection and transmission, motivating investigation into targeted early nasal treatments.

Antimicrobial photodynamic therapy (aPDT), photodynamic inactivation (PDI), or photodisinfection (PDF), uses light to activate otherwise non-toxic photosensitizers to inactivate pathogens and has shown efficiency against a wide range of microorganisms including gram-positive and-negative bacteria^8–10^, fungi^11^, parasites^12–16^ and viruses^17–27^. Treatment of viral infections using aPDT has a long clinical history dating back to research on herpes simplex virus^28,29^. It can rapidly (< 1 min) and effectively kill (inactivate > 5 logs) enveloped and non-enveloped viruses through high levels of oxidative stress produced at the viral membrane, viral capsid proteins and G-C base pairs of viral nucleic acids^18^. A stable form of methylene blue (MB) can be efficiently activated by a localized non-thermal red light (~ 670 nm) to produce excited singlet-state oxygen that is primarily responsible for the virucidal oxidative damage. Its application in human papillomavirus and related infections has been widely studied^20,21^. aPDT has also been used for treating blood products (Theraflex, Macopharma, France), including against HIV^30^, with additional studies on herpes, hepatitis A, B, and C^19^, adenoviruses, enteroviruses, cytomegalovirus and human parvovirus.

Nasal MB-aPDT was developed initially for peri-operative bacterial decolonization (Steriwave, Ondine Biomedical, BC, Canada) and has been clinically validated in high-risk surgical cases, leading to significantly reduced surgical site infection rates^31^. The treatment is innocuous and no significant adverse events have been noted in over 60,000 patients treated over 9 years^32^. As the SARS-CoV-2 virus initially colonizes the nose and can be transmitted by unknowing asymptomatic carriers, utilization of MB-aPDT as a low-risk and easy-to-apply local nasal therapy may be a valuable addition to the interventional arsenal to combat this pandemic. In evaluating its potential, it is important to quantify the level of SARS-CoV-2 inactivation not only in vitro but also in clinically-representative in vivo scenarios. This concept has been suggested by a number of authors and review articles^33–36^, however, Svyatchenko et al*.*^37^ were first to demonstrate by quantitative infectivity assay in limited number of samples (N = 16) that aPDT could be used to inactivate SARS-CoV-2 in vitro. More recent work in vitro by Arentz and von der Heide^38^ has confirmed the potential of aPDT against coronaviruses and specifically SARS-CoV-2. Subsequently, Schikora et al*.*^39^ reported the first aPDT clinical study against COVID-19. This trial utilized only clinical endpoints of disease progression and death rate, showing (i) reduction in the course of severe diseases, hospitalization and ICU admissions (2.6% vs. 19%), (ii) attenuation of disease progression (97% vs. 81%) and (iii) decreased mortality rate (0.7% vs. 3.3%). However, it provided no quantitative information on the photodynamic effect on the virus itself in the clinical setting. Hence, complementing and linking these reports, we present here: (i) a well-controlled in vitro study using MB-mediated aPDT against a model human betacoronavirus OC43 and against SARS-CoV-2, (ii) the first MB-aPDT clinical trial for early-stage Covid-19 with quantitative data on viral inactivation measured by both RT-qPCR and infectivity analyses and (iii) the feasibility of performing MB-aPDT on COVID-19 patients in a variety of health care and community settings.

## Results

### In vitro MB-aPDT in HCov-OC43

**Figure 1:**
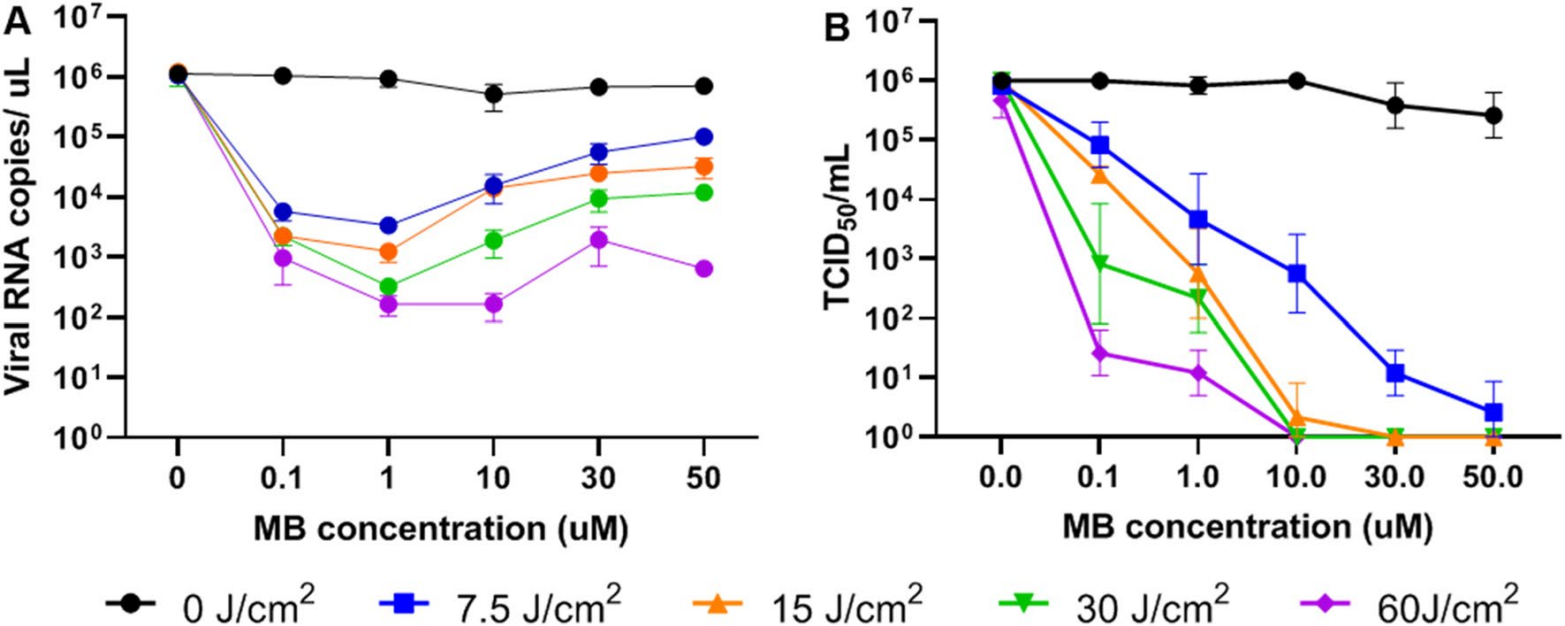
aPDT response in HCoV-OC43 inoculum. (**A**) RT-PCR analysis, showing the reduction in viral RNA copies per μl of total extracted RNA as a function of MB photosensitizer concentration for different light doses (coloured lines). (**B**) Corresponding infectivity measurements expressed as median tissue culture infection dose (TCID_50_). Data points are mean ± 1 standard deviation for n = 3–6 replicates. All aPDT protocols demonstrated significantly reduced viral RNA copies/μl and TCID compared to controls (light only, MB only, and no treatment groups, P < 0.05).

Due to initial limited availability of patient-derived samples containing SARS-CoV-2 virus and limited access to biosafety-level-3 laboratory facilities, dose-ranging studies with controlled light irradiation and photosensitizer delivery were carried out using the human betacoronavirus (HCoV-OC43), a clinically-relevant biosafety-level-2 coronavirus. RT-qPCR analysis showed a viral load reduction by a factor of up to 10^4^ (“4-Logs”) when aPDT was performed with 30 J cm^−2^ of 670 nm light exposure following incubation for 10 min in 1 μM MB and with 60 J cm^−2^ in 1–10 μM MB. However, it is known that viral inactivation by aPDT can induce damage that may not be detectable by RT-qPCR, resulting in under-estimation of the treatment effect, as seen, for example, in a recent study of MB-aPDT against Hepatitis-C^19^ in the setting of lung transplantation. This may be because aPDT only destroys the virus capside and/or causes RNA fragmentation which produces positive RT-PCR signals.that can falsely elevate RT-qPCR counts. Hence, infectivity assays were also performed to measure the capability of the virus to infect cells and replicate following treatment. This more clinically-relevant metric demonstrated up to 6 Logs of inactivation. The results of both assays are summarized in Fig. 1, showing the minimal effect of the photosensitizer alone (dark toxicity) and the systematic photosensitizer- and light-dose dependences. No significant differences were seen between the light-only, photosensitizer-only and no-treatment control groups. However, all groups treated with aPDT had significantly more viral inactivation compared to the controls (P < 0.05 for all).

### In vitro MB-aPDT in SARS-CoV-2

**Figure 2:**
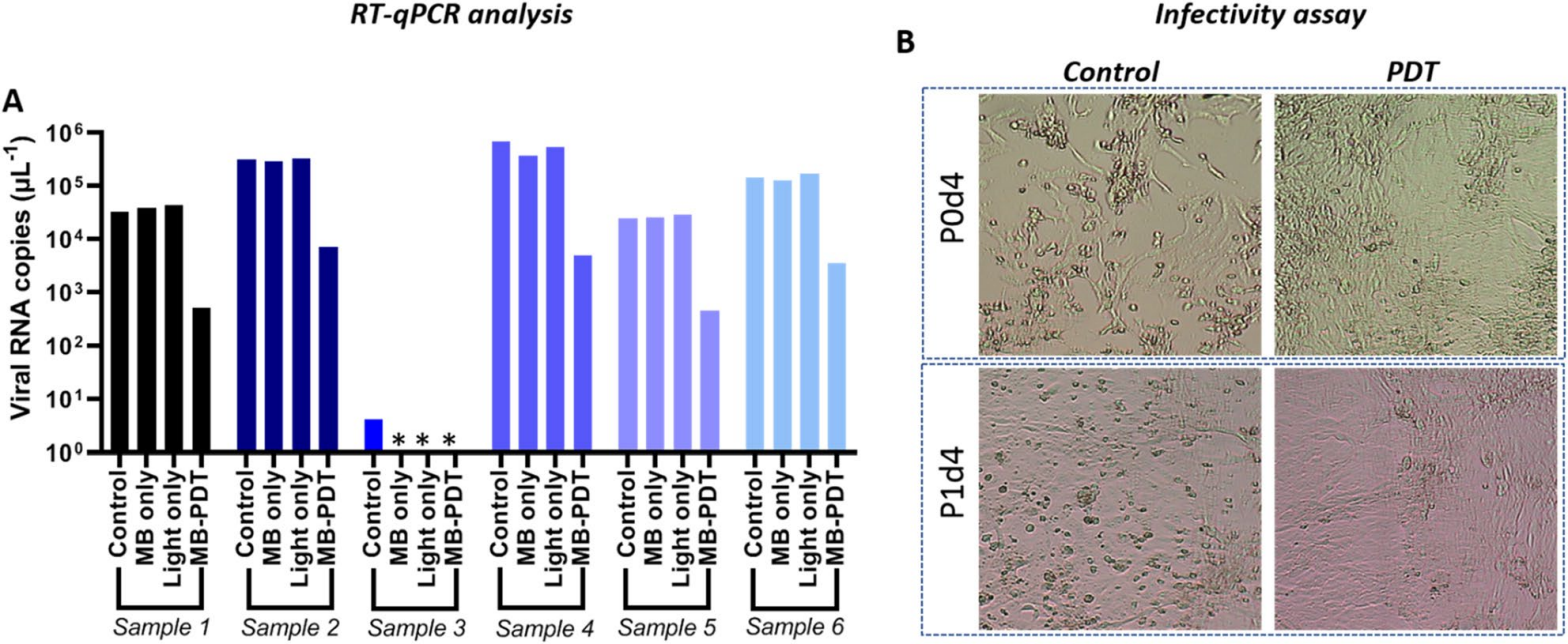
Effect of in vitro MB-aPDT treatment in SARS-CoV-2 patient-derived samples. (**A**) RT-qPCR analysis of 6 samples treated with MB-aPDT (10 min incubation at 10 μM and 30 J cm^−2^), together with control untreated and light-only or MB-only controls. *Samples with undetectable level of SARS-CoV-2. (**B**) Representative cytopathic effect (CPE) slides for aPDT-treated and control untreated samples #1 and #4. No viral load can be seen post-aPDT. In P0d4 and P1d4, P represents the cell passage number and d the day of analysis. Significant reductions were observed between the aPDT and control groups in both viral RNA copies/μl and infectivity (P < 0.05).

Six patient-derived samples containing SARS-CoV-2 in universal transport media were treated with MB-aPDT. A significant reduction in the viral load of up to ~ 2 Logs as measured by RT-qPCR was observed in all samples treated with MB + light (Fig. 2A) compared to the control groups (P < 0.05 for all). No difference was observed in the MB-only and light-only controls, with the exception of one sample (#3) in which only a low viral load was detectable pre-treatment but became undetectable after treatment with either MB or light alone. Samples #1 and #4 were also analyzed for infectivity and in both cases the measurable infectivity of the untreated samples was eliminated (Fig. 2B). The lack of infectivity reflects the inability of the virus to infect cells (loss of its cytopathic effect, CPE), preventing its replication, further suggesting that the post-treatment RT-qPCR signal does not represent functional virus.

The raw data used in the in vitro assessment are available from the corresponding author upon reasonable request.

### Clinical trial

**Figure 3:**
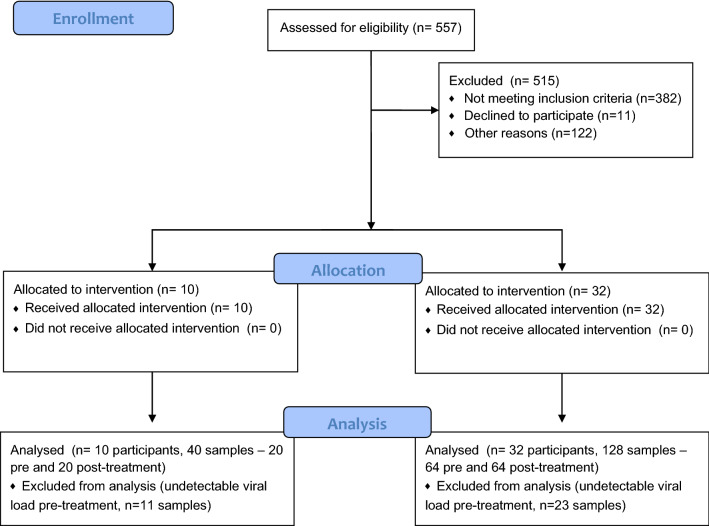
Flow diagram.

Of the 42 patients enrolled in the study with a positive Covid-19 diagnosis (age 14–94 y, 20 male, 18 female, 4 no sex stated) 18 were symptomatic (flow diagram, Fig. 3). The study was conducted within 6 different settings: ICU (N = 1), in-patient ward (N = 6), out-patient Covid clinic (N = 5), in subject’s car (N = 17), in-patient rehabilitation hospital (N = 10) or on patient’s home front porch (N = 3). The procedures were performed by a surgeon (N = 11), a surgical resident (N = 18), a ward nurse (N = 4) and an ER nurse (N = 7). All had received training in the aPDT procedure, including pre- and post-treatment nasal swabbing and application of the MB and light. The treatment was simple to administer in all environments and well tolerated by all patients, with no discomfort, complications or side effects reported. The total treatment time, including pre- and post-treatment nasal swabbing and MB-aPDT application was 10–15 min. Oxygen was paused briefly in one ICU patient to allow the procedure. Two technical problems arose when using the laser outside its recommended operating environment. First, in performing multiple treatments initially with the laser inside a sterile bag, overheating caused a pause in the light delivery. This was resolved by removing the bag and sterilizing the laser between sessions. Second, with one subject treated out-of-doors in winter the low temperature affected the operation of the laser and light delivery had to be paused for 5 min. No other technical problems were encountered.

**Figure 4:**
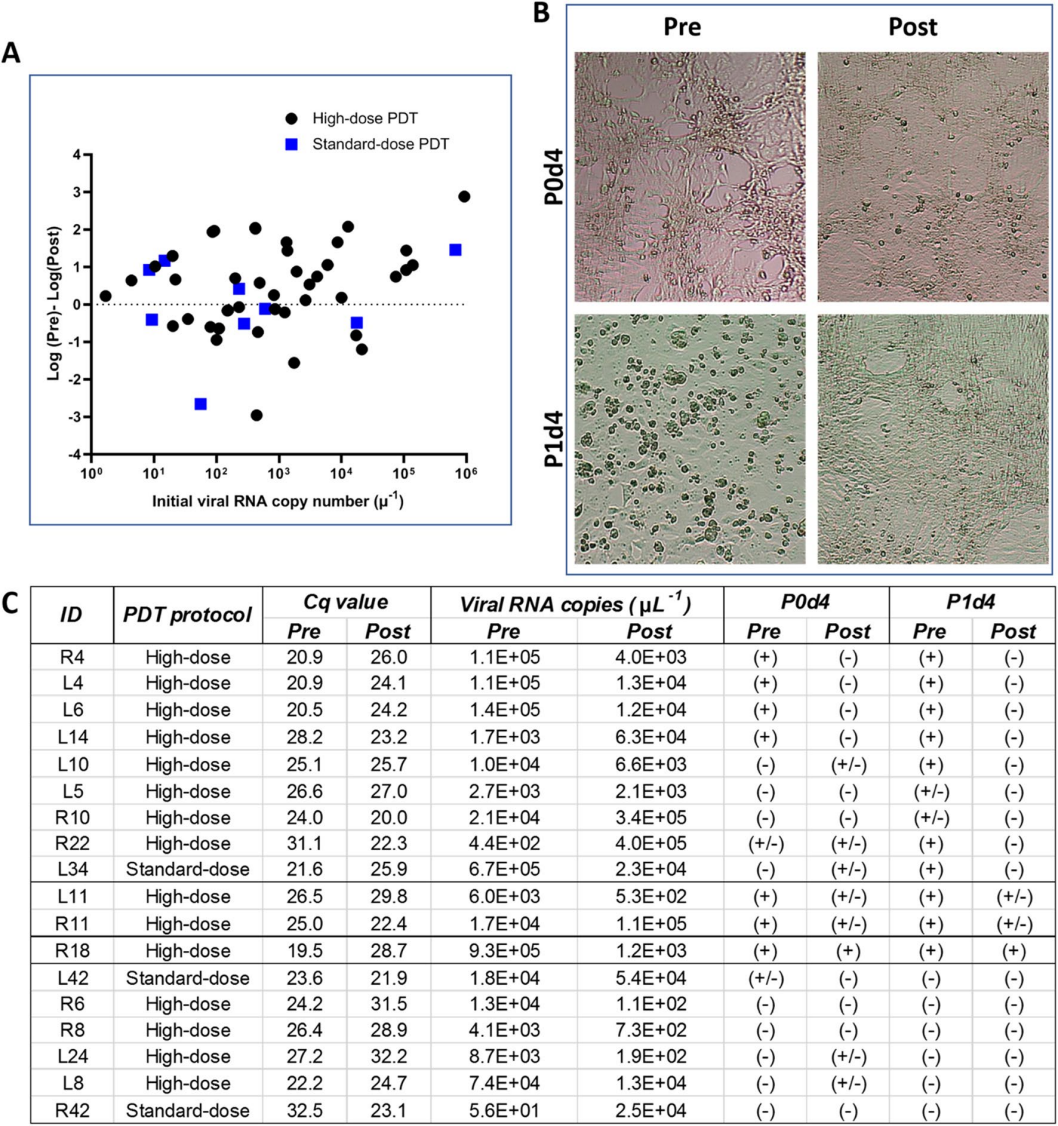
Effect of MB-aPDT on SARS-CoV-2 infectivity in clinical samples. (**A**) Change in viral RNA copies following nasal aPDT vs. pre-treatment load in the same nostril, as measured by RT-qPCR. Black circle 72 J cm^−2^, blue square 36 J cm^−2^. (**B**) Representative microscope images of inoculated Vero-76 cells. The cytopathic effect seen in the pre-treatment samples is not observed post-aPDT. (**C**) Individual clinical sample results, showing Cq values and viral RNA copy number per μL from RT-qPCR, together with the P0d4 and P1d4 infectivity results. The samples are labeled as in the Supplementary material that presents the complete data set.

Figure 4A summarizes the pre- and post-treatment PCR results from the subjects treated with standard (72 J cm^−2^, N = 10 participants, 40 samples pre + post-treatment) and high-dose aPDT (144 J cm^−2^, N = 32 participants, 128 samples pre + post-treatment), plotting the data for each nostril independently. Forty percent (N = 34/84) of the samples were negative for SARS-CoV-2 in the nasal pre-treatment swabs. Although these patients were all diagnosed as COVID-19+ through clinical nasopharyngeal testing, the delay between their initial COVID+ test results and recruitment into the study may have altered the detectable viral load in the anterior nostrils. aPDT resulted in viral load reduction in 64% (27/42, P = 0.07) and 44% (4/9, P = 0.57) of treated nostrils using the high and standard aPDT doses, respectively. A reduction > 90% in viral load was seen in 38% (16/42) of the nostrils treated with high-dose aPDT.

Although RT-qPCR is known to yield limited accuracy with respect to treatment response following aPDT, it was an important measure to quantify the presence of SARS-CoV-2 in pre-treatment swabs before proceeding to the infectivity analysis, since high pre-treatment viral load increases the success of the infection assay.

The results of the infectivity assay are shown in Fig. 4B,C. Due to limited Level-3 facility access, representative samples were selected from those with Cq < 32 pre-treatment (n = 18). As there were limited viral load levels in some of these samples, the supernatant was removed from the P0d4 culture and added to a new ready-to-use Vero-76 culture tube for a second CPE evaluation (P1d4). Enabling the virus to replicate in this way increases the accuracy of the assay.

For the nostrils treated with 144 J cm^−2^, positive infectivity was found in 47% (7/15) of samples at the evaluation following 4d culture (P0d4). At this timepoint, reduced or undetectable infection was seen in 86% (6/7 P < 0.05,) of the samples following aPDT. On the second evaluation at P1d4, 66% (10/15) of samples were positive pre-treatment and aPDT treatment reduced the infectivity in 90% (9/10, P < 0.05) of these samples and completely inactivated the virus (within the sensitivity of the assay) in 70% (7/10, P < 0.05). Four of the pre-treatment samples became more infectious after the 4d incubation, whereas none of the post-treatment samples did so. Although only a small cohort of patients received the lower light dose, one of the three pre-treatment samples and none of the post-treatment samples showed infectivity at P1d4.

These infectivity data confirm that RT-qPCR does indeed underestimate the efficacy of aPDT for SARS-CoV-2: for example, sample L14 had Cq = 28.2 pre-treatment and a higher viral load (Cq = 23.2) post-aPDT, whereas the corresponding post-aPDT infectivity assay showed an undetectable level of active virus.

All data generated and analysed within the clinical trial are included in the published article and its supplementary material.

## Discussion

Photodisinfection with methylene blue and non-thermal light is a mechanistically-robust, safe, Health Canada-approved in vivo clinical procedure that has been successfully utilized to kill both bacterial and viral pathogens, including in the nares^32^. In general, aPDT in a number of settings has shown high efficacy in viral inactivation; it is low-risk, non-toxic, minimally-invasive, rapid, repeatable and easy to use with minimal training. We have demonstrated here that aPDT is efficient in inactivating human coronavirus in vitro and in the clinical scenario based on established and quantitative microbiological endpoints. This is the first study demonstrating via objective and quantitative clinical viral load reduction measurements that MB-aPDT is a safe, easily delivered, effective front-line technique that can reduce local SARS-CoV-2 viral load. Noting that aPDT can lead to RNA fragmentation and falsely elevate RT-qPCR counts, this study also draws attention to the need to utilize infectivity as the viral response metric in studies of photodynamic treatments, supporting earlier findings in Hepatitis-C in the setting of organ transplantation^19^.

As indicated above, Schikora et al*.*^39^ reported the first aPDT clinical study against COVID-19, using clinical endpoints that showed positive responses but without direct virological measurements or statistical analysis. Recent work by Svyatchenko et al*.*^37^ employed an infectivity assay in Vero E6 cells to show that aPDT could effectively kill SARS-CoV-2 in vitro, and that cell viability was not compromised, but used only two different light doses and photosensitizer concentrations and a small sample size. Arentz and von der Heide^38^, similarly showed the potential of aPDT on a bovine coronavirus (BCoV) and SARS-CoV-2 in vitro using low doses of MB and at a range of light doses supplied by a laser or LED source. Here, systematic light and photosensitizer dose over a full range was used, with the responses measured by both RT-qPCR and infectivity assays. aPDT was shown to inactivate HCoV-OC43 by up to 6-Logs in vitro, as measured by RT-qPCR and by infectivity assay in HCT-8 cells. Furthermore, aPDT performed in vitro on samples from COVID-19 patients showed significant reduction in Cq values and 100% viral inactivation measured by the infectivity assay. The fact that aPDT was equally effective in two different human coronaviruses highlights the non-specific effect of the therapy and, hence, its potential in the fight against both current and future coronavirus variants.

The clinical trial in subjects testing positive for SARS-CoV-2 revealed that, immediately after aPDT, 78% of the nasal-swab samples had reduced viral load as measured by RT-qPCR, which amplifies and quantifies specific RNA gene sequences. An important caveat, however, is that the reactive oxygen species generated during aPDT primarily damage the viral envelope, exposing the RNA to the environment that, in turn, can fragment the RNA and so give falsely high PCR counts. This effect was likely seen in some of the clinical samples in which a large increase in Cq was observed after aPDT but the post treatment samples were non-infective. We included samples R22 and R42 in the infectivity assay (which, despite large increases in viral load measured by RT-qPCR, showed limited to no infectivity post treatment) specifically to confirm the limitations of RT-PCR in determining the impact of aPDT. Nevertheless, RT-qPCR testing was still useful, as it guaranteed the presence of SARS-CoV-2 in the samples, so that negative pre-treatment patients could be censored from the subsequent infectivity testing, given the limited biosafety-level-3 resources. The much more important finding was that the infectivity was reduced in 90% of the samples following intranasal aPDT and was undetectable in 70% of them using a standardized assay in Vero-76 cells. Access to, and resources limitations at, BSL3 facilities are also improving which will enable more comprehensive infectivity testing in future trials.

A practical challenge in this clinical study in the context of a rapidly-evolving pandemic was to access and recruit patients with an early positive molecular test for COVID-19. SARS-CoV-2 is found primarily in the upper respiratory tract in the first 5 days after infection, which would be the ideal time for nares (or more extended upper oral-nasopharyngeal) aPDT. We recruited patients with positive nasopharyngeal COVID-19 diagnostic swabs but the timing of infection was unknown, so that some subjects recruited into the study had undetectable RT-qPCR levels of SARS-CoV-2 based on the anterior-nares swabs. Additional challenges with recruitment during a pandemic and the high variability in viral load between patients (and even between nostrils in the same patient) motivated a repeated-measures study design comparing pre- and post-treatment swabs, rather than utilizing a separate non-treated (or sham) control group. Thus, pre-treatment swabs served as patient (and nostril) specific controls and we report only viral load reduction and inactivation, rather than attempting to evaluate any clinical or longer-term outcomes, for which a separate untreated control group would be necessary. This is a recommended focus of future studies, but may present challenges with respect to feasibility in attaining a sufficient sample size because of the difficulty in obtaining patient consent where one arm represents no treatment.

While these practical limitations impacted the sample size available for this study, the knowledge gained is informative with respect to how such clinical trials need to be designed and executed in the future. With current widespread access to rapid testing, recruitment within a test-and-treat setting would facilitate trial enrollment, allowing for aPDT treatment immediately after the infection is first confirmed. In addition to demonstrating the potential of MB-aPDT to reduce and inactivate Covid-19 intra-nasally, this proof-of-principle clinical study has demonstrated the feasibility of delivering aPDT in diverse settings, which is critical for translation to clinical practice, including: ICUs, outpatient clinics, long-term care facilities, homes and vehicles. The minimal requirements for technical infrastructure and specialized personnel are important factors in the future dissemination of this modality, including in remote and low-resource regions.

Due to sample size, we cannot conclude whether reducing the treatment time from 8 min to the 4 min as routinely used for peri-surgical bacterial decolonization (144 vs*.* 72 J cm^–2^ light dose) in order to facilitate treatment delivery would result in reduced efficacy. The longer treatment time was well tolerated in all subjects in the high-dose group. Nevertheless, the data do indicate a level of efficacy at the lower light dose, motivating further investigation to determine the optimal clinical light dose/treatment time for aPDT in the context of SARS-CoV-2.

Although not assessed here, a further potential use of aPDT is to protect acutely-exposed healthcare workers (respiratory therapists, intensivists, anesthesiologists, emergency and ICU staff). Such "nose hygiene" could ultimately be used as a first line of defense against SARS-CoV-2 (or other respiratory pathogens) by inactivating virus in the nares before infection can occur: SARS-CoV-2 is reportedly present in the anterior nares for 10–12 days prior to more widespread pulmonary dissemination^6^. This strategy could also limit transmission to at-risk healthcare workers from SARS-CoV-2 patients undergoing aerosol-generating procedures. The potential benefit is exemplified by the devastating effects on clinical specialists in China exposed to SARS-CoV-2 patients during intubation. Reports of viable SARS-CoV-2 found in aerosols for more than 3 h highlights the importance of this issue^40,41^.

Additional roles for aPDT in limiting the spread of SARS-CoV-2 beyond the hospital environment can also be envisaged, e.g., prophylactically in individuals working with high-risk populations, such as in long-term care facilities. In this context it is important to note that numerous studies have shown no evidence of aPDT-induced resistance^42–46^, so that repeat photodisinfection can be performed without loss of efficacy. Moreover, the mechanism of aPDT viral inactivation is not protein-specific, suggesting that it should be equally effective on new variants: this is currently under investigation.

Finally, we are working on minimally-invasive photosensitizer and light delivery methods to deliver aPDT beyond the nares to include the nasopharynx and oral cavity. This approach will be evaluated in future trials to determine the potential benefit in reducing the time to recovery from SARS-CoV-2 through lowering of the total viral load in the upper respiratory tract, thereby allowing the native immune system to combat the virus and minimize the risk of a cytokine storm. Such broad-spectrum treatment of a larger area of the upper airway represents a potential low-cost option for viral, bacterial and fungal respiratory infections.

## Conclusions

MB-aPDT was found to be effective in inactivating human coronavirus, HCoV-OC43 and SARS-CoV-2, in vitro measured by RT-qPCR and infectivity assays, reaching up to 6 Logs of inactivation. In a proof-of-principle clinical trial, MB-aPDT was investigated as a therapy for early stages of COVID-19. RT-qPCR was able to identify the presence of SARS-CoV-2 in the samples, although it was not accurate for measuring the aPDT response. Cell-infectivity measurements revealed a viral reduction in 90% of the samples and caused complete inactivation in 70% after a single session of aPDT. Furthermore, this study has shown the feasibility of aPDT to locally reduce and inactivate COVID-19 in its early stages with minimal infrastructure in varied environments. To our knowledge, this is the first aPDT clinical trial for COVID-19 with virological quantification via RT-qPCR and infectivity assay. These promising results indicate the potential of aPDT as a novel tool against COVID-19, motivating future larger trials that would include both short- and longer-term clinical outcomes. If successful, this advance would be important not only in the current SARS-CoV-2 pandemic, but also to other future viral or bacterial transmissible diseases where a safe and inexpensive local/regional non-specific treatment may be applied rapidly.

## Materials and methods

### In vitro study—Betacoronavirus OC43

#### Virus propagation

The Human Betacoronavirus (HCoV-OC43, VR-1558) and the human cell line HCT-8 (HRT-18) were purchased from the American Type Culture Collection. The cells were cultured in RPMI supplemented with 10% fetal bovine serum and kept in an incubator at 37 °C and 5% CO_2_. They were transferred to T25 flasks for virus propagation and infected at 80–90% confluence, as follows. The cells were washed twice with PBS. The virus, diluted in 1 mL of RPMI (without phenol red) and supplemented with 2% FBS, was added to the cells, which were then held at 33 °C and 5% CO_2_. The supernatant was collected when the cytopathic effect (CPE) progressed through 80% of the monolayer. The viral load in the supernatant was quantified by RT-PCR and viral titration using standard methods^47^.

### MB-aPDT

10^6^ TCID_50_ (median Tissue Culture Infectious Dose) in 500 μL of RPMI 1640 (Roswell Park Memorial Institute Medium) supplemented with heat inactivated fetal bovine serum without phenol red was added to 24-well plates in triplicate. Methylene Blue (Sigma Aldrich) at concentrations of 0.1, 1, 10, 30 or 60 μM was added and the plates were incubated for 10 min prior to light irradiation, which utilized a custom-built LED (Light-Emitting Diodes) array with 660 nm peak wavelength and an output power density of 30 mW cm^−2^. A range of light energy densities was investigated (7.5, 15, 30 and 60 J cm^−2^) by varying the irradiation time from 4 to 33 min. Controls included light-only, MB-only and no treatment. All conditions were replicated in triplicate (180 samples in total). Immediately after treatment, the samples were serially diluted and frozen for RT-PCR and infectivity analyses. After adding MB, all cells were kept under minimal ambient light.

#### RT-PCR analysis

RNA was isolated using the RNAdvance Viral XP kit (Beckman Coulter) as per the manufacturer’s protocol. HCoV-OC43 RNA was analyzed by RT-qPCR using 10 µl reactions with a 2× One-Step RT-PCR Master Mix (Norgen Biotek), 2.5 µl of RNA extract and primer/probe sets for the membrane protein gene (For: ATGTTAGGCCGATAATTGAGGACTAT, 300 nM; Rev: AATGTAAAGATGGCCGCGTATT, 300 nM; Probe: Cy5-CATACTCTGACGGTCACAAT, 200 nM)^48^ or N gene (For: CGATGAGGCTATTCCGACTAGGT, 450 nM; Rev: CCTTCCTGAGCCTTCAATATAGTAACC, 450 nM; Probe: HEX-TCCGCCTGGCACGGTACTCCCT, 100 nM)^49^. Samples were analyzed on a BioRad CFX384 real-time PCR system with the following settings: 50 °C for 30 min, 95 °C for 3 min, then 45 cycles of 95 °C for 3 s and 60 °C for 30 s, followed by fluorescence detection. To quantify viral copy number, standard curves were generated using SARS-CoV-2 or HCoV-OC43 synthetic RNA standards (Twist Biosci).

#### Infectivity assay

50,000 HCT-8 cells were cultured overnight in 96-well plates with RPMI (5% FBS). A virus titration was then carried out as follows. Ten microliters of HCoV-OC43 samples treated with MB-aPDT, and the corresponding controls, were serially diluted (range 10^–1^–10^–6^) and added to each well containing 90 μL RPMI media (2% FBS). The plates were kept in an incubator at 33 °C and 5% CO_2_ for 4 days. They were then washed twice with PBS and fixed with cold methanol for 15 min. An immunofluorescence assay was carried out as described by Owczarek et al*.*^50^. For this, the fixed cells were washed twice with PBS and permeabilized with 0.5% Triton X-100 for 10 min at room temperature. The permeabilizer was removed and the cells were incubated overnight with 5% bovine serum albumin (BSA). After washing with PBS, the primary anti-HCoV-OC43 antibody (MAB9012, Merck) diluted 1:1000 in 3% BSA was added for 2 h. The cells were then incubated with a secondary AlexaFluor-labeled goat anti-mouse IgG antibody (Thermo Fisher Scientific) for 1 h and observed in a confocal fluorescence microscope (488 nm excitation, 530 nm detection).

### In vitro study—SARS-Cov-2

#### MB-aPDT treatment

Patient-derived SARS-CoV 2 inoculum (positivity confirmed by RT-PCR) was seeded in 24-well plates (50 μL/well) with 400 μL of DMEM without phenol red. MB was added to a final volume of 500 ml, giving a concentration of 10 μM. Irradiation of each plate was carried out after 10 min incubation using the same LED source to deliver 30 J cm^−2^. Controls included light-only, MB-only and no treatment.

#### PCR analysis

SARS-CoV-2 RNA (Orf1ab) was isolated as described above and analyzed by RT-qPCR using the TaqMan-based 2019-nCoV: Real-Time Fluorescent RT-PCR kit from BGI as previously described^51^. Briefly, 10 µl reaction volumes containing 2.5 µl of RNA extract were analyzed using a BioRad CFX384 real-time PCR system according to the manufacturer’s recommended cycling conditions.

#### Infectivity assay

Samples quantified by RT-qPCR with a Cq value < 32 were sent to a Biosafety-level-3 lab (Roy Romanow Provincial Laboratory, Saskatchewan Health Authority, Regina, Canada) for infectivity assay. For this, the viral inoculum was added to ready-to-use Vero-76 culture tubes and kept in an incubator at 37 °C and 5% CO_2_ for 1 h. One mL of RM-02 REFEED medium (Quidel [Diagnostic Hybrids] 10-320500) with 2% FBS, antibiotics and TPCK-treated trypsin (1.0 µg/mL for samples with a Cq value ≤ 25, or 16 µg/mL for samples with Cq value > 25; ThermoFisher Scientific) was then added to each tube and the cells were monitored for CPE (P0d4). On day 4 post-inoculation, 100 µl of the supernatant was removed and added to a new ready-to-use Vero-76 culture tube for the second CPE (P1d4) evaluation. This allowed the virus to replicate in the first assay and increase its infection capability in the second. It is also important for determining that the CPE seen is truly from viral replication and not sample related. This approach is required for samples with low viral load and increases the analytic accuracy.

### Clinical trial

#### Study design

This protocol was approved by the Sunnybrook Health Sciences Centre Research Ethics Board (#2069) and registered at ClinicalTrials.Gov, NCT04615936, NasalPDF0001) on 2020/11/04.

All the procedures were carried out in accordance with the ethical standards of the responsible committee of human experimentation (institutional and national). Informed consent was obtained from all subjects. For participants under 18 years of age, the consent was obtained from a parent and/or legal guardian. Participants were recruited between October 2020 and May 2021. The study protocol is provided in Supplementary Material. All participants were 14 years of age or older, presented with symptomatic or asymptomatic SARS-CoV-2, and had positive molecular testing for SARS-CoV-2 within the previous < 11 days (4.2 ± 3.4 days). The study utilized a repeated-measures design in which pre-treatment swabs served as patient (nostril) specific controls and were compared against viral load measured via RT-qPCR and infectivity testing post-treatment.

#### Treatment

**Figure 5:**
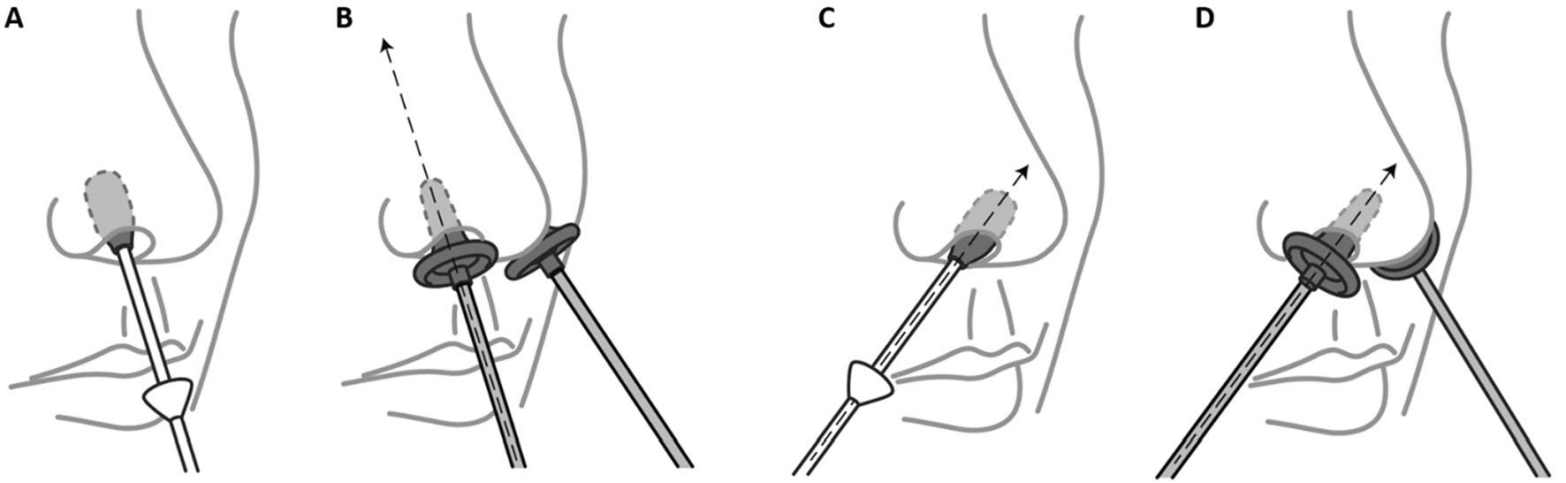
Illustration of the photodisinfection procedure in the anterior nares. (**A**) Photosensitizer application, (**B**) illumination pointing towards the forehead, (**C**) light applicator repositioning emphasizing the anterior nostril, (**D**) illumination of the anterior nostrils. This illustration was designed by Ondine Medical Inc.

The Health Canada-approved Steriwave system (Ondine Biomedical, Vancouver, BC, Canada) was utilized to deliver MB-aPDT to the anterior nares^20^. After informed consent, participants (N = 42) were asked to blow their nose to reduce nose secretion. An initial swab was performed for deeper cleaning. A second swab was then used to collect the pre-treatment sample (Step 1, Fig. 5). MB (0.01%) was applied using a swab to each nostril (Step 2, Fig. 5). An applicator connected to a dual channel fiberoptic applicator connected to a 670 nm diode laser (300 mWcm^-2^) was placed simultaneously into both nostrils (Step 3, Fig. 5) pointing to midline of the nose (Fig. 5B). After irradiation, MB was re-applied and a second light dose was given with the applicator positioned pointing towards the nares anterior pocket by changing the angle of the nasal applicator (Fig. 5D). Immediately post-treatment, a third swab was collected (Step 4, Fig. 5). The pre and post treatment samples were immediately frozen in saline and stored at −80 °C. RT-PCR and infectivity analyses were performed following the same protocol as in the in vitro studies above. Two light doses were investigated: a high-PDT dose, 144 J cm^−2^ (N = 32) and the standard dose of 72 J cm^−2^ that is used clinically for pre-surgical nasal decolonization (N = 10).

### Statistical analysis

Statistical analysis was performed using GraphPad Prism 9.3.1. The in vitro studies were compared using 2-way ANOVA. For the clinical trial, the viral load pre and post-treatment in each nostril was compared using the Wilcoxon matched-pairs signed-rank test. The binomial test was used to analyze the infectivity assay data. In all cases, a value of P < 0.05 was considered statistically significant.

## Supplementary Information

Supplementary Table S1.
