# Supplementary material for: Translational feasibility and efficacy of nasal photodynamic disinfection of SARS-CoV-2
Source: Sci Rep. 2022 Aug 24;12:14438. doi: 10.1038/s41598-022-18513-0 (PMC9400568; doi:10.1038/s41598-022-18513-0)
Supplement: Supplementary file 1 — Supplementary Table S1. [file 41598_2022_18513_MOESM1_ESM.docx]

**Translational Feasibility and Efficacy of Nasal Photodynamic Disinfection of SARS-CoV-2**

Layla Pires^1^, Brian C. Wilson^1,2^, Rod Bremner^2,3^, Amanda Lang^4^, Jeremie Larouche^2,5^, Ryan McDonald^4^, Joel D. Pearson^3^, Daniel Trcka^3^, Jeff Wrana^2,3^, James Wu^2,5^, Cari Whyne^2,5*^

^1^ University Health Network

^2^ Faculty of Medicine, University of Toronto

^3^ Lunenfeld-Tanenbaum Research Institute, Mount Sinai Hospital

^4^ Roy Romanow Provincial Laboratory, Saskatchewan Health Authority

^5^ Holland Bone and Joint Program, Sunnybrook Research Institute

*Corresponding author:

Cari Whyne

2075 Bayview Avenue S620

Toronto ON Canada M4N3M5

Phone 416 480 5056

[cari.whyne@sunnybrook.ca](mailto:cari.whyne@sunnybrook.ca)

**Supplementary Material**

*Table S1. Summary of the RT-qPCR data from swabs of the anterior nares pre and post PDF from 42 patients with a recent Covid+ diagnosis.. R and L refer to right and left nostrils. ND means non-detectable viral load. The high dose represents a light delivery of 144 Jcm^-2^, whereas the standard dose is 72 Jcm^-2^. The reduction rate data shown in red reflect a lower viral load (higher Cq value) following PDF.*

| *Patient ID* | *Nostril* | *Cq value* | | *CoV-2 quant (copies/ul in RNA)* | | | *Protocol* |
| --- | --- | --- | --- | --- | --- | --- | --- |
|  |  | *Pre* | *Post* | *Pre* | *Post* | *Reduction rate* |  |
| 1 | R | ND | ND | ND | ND | * | High-dose |
|  | L | ND | ND | ND | ND | * |  |
| 2 | R | ND | ND | ND | ND | * | High-dose |
|  | L | ND | ND | ND | ND | * |  |
| 3 | R | ND | 36.03 | ND | 5.8E+00 | * | High-dose |
|  | L | 35.12 | ND | 1.0E+01 | ND | -100 |  |
| 4 | R | 20.86 | 25.96 | 1.1E+05 | 4.0E+03 | -96 | High-dose |
|  | L | 20.86 | 24.12 | 1.1E+05 | 1.3E+04 | -88 |  |
| 5 | R | 29.17 | 31.23 | 5.0E+02 | 1.3E+02 | -74 | High-dose |
|  | L | 26.58 | 26.98 | 2.7E+03 | 2.1E+03 | -23 |  |
| 6 | R | 24.16 | 31.54 | 1.3E+04 | 1.1E+02 | -99 | High-dose |
|  | L | 20.49 | 24.21 | 1.4E+05 | 1.2E+04 | -91 |  |
| 7 | R | 29.29 | 26.66 | 4.6E+02 | 2.5E+03 | 448 | High-dose |
|  | L | 27.76 | 27.01 | 1.2E+03 | 2.0E+03 | 63 |  |
| 8 | R | 26.43 | 28.95 | 4.1E+03 | 7.3E+02 | -82 | High-dose |
|  | L | 22.17 | 24.67 | 7.4E+04 | 1.3E+04 | -82 |  |
| 9 | R | ND | ND | ND | ND | * | High-dose |
|  | L | ND | ND | ND | ND | * |  |
| 10 | R | 24.01 | 19.95 | 2.1E+04 | 3.4E+05 | 1481 | High-dose |
|  | L | 25.10 | 25.73 | 1.0E+04 | 6.6E+03 | -35 |  |
| 11 | L | 26.47 | 29.83 | 6.0E+03 | 5.3E+02 | -91 | High-dose |
|  | R | 25.02 | 22.40 | 1.7E+04 | 1.1E+05 | 567 |  |
| 12 | L | ND | 34.36 | ND | 2.0E+01 | * | High-dose |
|  | R | 32.00 | 29.97 | 1.1E+02 | 4.8E+02 | 338 |  |
| 13 | L | 31.53 | 31.03 | 1.5E+02 | 2.2E+02 | 44 | High-dose |
|  | R | 30.15 | 36.65 | 4.2E+02 | 3.7E+00 | -99 |  |
| 14 | L | 28.17 | 23.23 | 1.7E+03 | 6.3E+04 | 3503 | High-dose |
|  | R | ND | ND | ND | ND | * |  |
| 15 | L | 34.35 | ND | 2.0E+01 | ND | -100 | High-dose |
|  | R | ND | 36.58 | ND | 3.9E+00 | * |  |
| 16 | L | 32.05 | 29.12 | 1.0E+02 | 8.8E+02 | 740 | High-dose |
|  | R | 33.55 | 32.33 | 3.5E+01 | 8.6E+01 | 142 |  |
| 17 | L | 34.21 | 36.31 | 2.2E+01 | 4.8E+00 | -78 | High-dose |
|  | R | ND | 33.10 | ND | 4.9E+01 | * |  |
| 18 | L | 31.17 | 33.29 | 2.0E+02 | 4.3E+01 | -79 | High-dose |
|  | R | 19.51 | 28.66 | 9.3E+05 | 1.2E+03 | -100 |  |
| 19 | L | 32.32 | ND | 8.6E+01 | ND | -100 | High-dose |
|  | R | 27.39 | 29.09 | 3.1E+03 | 9.0E+02 | -71 |  |
| 20 | L | 28.56 | 33.83 | 1.3E+03 | 2.9E+01 | -98 | High-dose |
|  | R | 36.44 | ND | 4.4E+00 | ND | -100 |  |
| 21 | L | ND | ND | ND | ND | * | High-dose |
|  | R | ND | ND | ND | ND | * |  |
| 22 | L | 29.16 | 31.77 | 1.9E+03 | 2.5E+02 | -87 | High-dose |
|  | R | 31.06 | 22.29 | 4.4E+02 | 4.0E+05 | 89212 |  |
| 23 | L | ND | ND | ND | ND | * | High-dose |
|  | R | ND | ND | ND | ND | * |  |
| 24 | L | 27.22 | 32.16 | 8.7E+03 | 1.9E+02 | -98 | High-dose |
|  | R | 29.60 | 33.79 | 1.4E+03 | 5.3E+01 | -96 |  |
| 25 | L | 30.19 | 29.83 | 8.7E+02 | 1.1E+03 | 32 | High-dose |
|  | R | 30.24 | 30.99 | 8.3E+02 | 4.7E+02 | -44 |  |
| 26 | L | ND | ND | ND | ND | * | High-dose |
|  | R | ND | ND | ND | ND | * |  |
| 27 | L | 35.03 | 33.35 | 2.0E+01 | 7.5E+01 | 267 | High-dose |
|  | R | 33.29 | 31.48 | 7.8E+01 | 3.2E+02 | 308 |  |
| 28 | L | 38.25 | ND | 1.7E+00 | ND | -100 | High-dose |
|  | R | ND | ND | ND | ND | * |  |
| 29 | L | 33.08 | ND | 9.2E+01 | ND | -100 | High-dose |
|  | R | ND | ND | ND | ND | * |  |
| 30 | L | ND | 24.82 | ND | 5.6E+04 | * | High-dose |
|  | R | ND | ND | ND | ND | * |  |
| 31 | L | 31.91 | 31.69 | 2.3E+02 | 2.7E+02 | 18 | High-dose |
|  | R | 35.07 | ND | 2.0E+01 | ND | -100 |  |
| 32 | L | ND | ND | ND | ND | * | High-dose |
|  | R | ND | ND | ND | ND | * |  |
| 33 | L | ND | ND | ND | ND | * | Standard-dose |
|  | R | ND | 32.92 | ND | 1.1E+02 | * |  |
| 34 | L | 21.61 | 25.94 | 6.7E+05 | 2.3E+04 | -97 | Standard-dose |
|  | R | 31.67 | 30.15 | 2.8E+02 | 9.0E+02 | 224 |  |
| 35 | L | ND | ND | ND | ND | * | Standard-dose |
|  | R | ND | ND | ND | ND | * |  |
| 36 | L | ND | ND | ND | ND | * | Standard-dose |
|  | R | 35.31 | 33.87 | 9.2E+00 | 2.4E+01 | 154 |  |
| 37 | L | ND | ND | ND | ND | * | Standard-dose |
|  | R | ND | ND | ND | ND | * |  |
| 38 | L | 30.34 | 31.83 | 2.3E+02 | 8.8E+01 | -62 | Standard-dose |
|  | R | 28.86 | 28.45 | 6.0E+02 | 7.8E+02 | 30 |  |
| 39 | L | 34.59 | ND | 1.5E+01 | ND | -100 | Standard-dose |
|  | R | 35.47 | ND | 8.4E+00 | ND | -100 |  |
| 40 | L | ND | ND | ND | ND | * | Standard-dose |
|  | R | ND | ND | ND | ND | * |  |
| 41 | L | ND | ND | ND | ND | * | Standard-dose |
|  | R | ND | ND | ND | ND | * |  |
| 42 | L | 23.64 | 21.9 | 1.8E+04 | 5.4E+04 | 208 | Standard-dose |
|  | R | 32.53 | 23.08 | 5.6E+01 | 2.5E+04 | 44910 |  |
